# Functional Genomic Validation of the Roles of *Soluble Starch Synthase IIa* in *Japonica* Rice Endosperm

**DOI:** 10.3389/fgene.2020.00289

**Published:** 2020-04-02

**Authors:** Vito M. Butardo Jr., Jixun Luo, Zhongyi Li, Michael J. Gidley, Anthony R. Bird, Ian J. Tetlow, Melissa Fitzgerald, Stephen A. Jobling, Sadequr Rahman

**Affiliations:** ^1^CSIRO Agriculture and Food, Canberra, ACT, Australia; ^2^Department of Chemistry and Biotechnology, Faculty of Science, Engineering and Technology, Swinburne University of Technology, Hawthorn, VIC, Australia; ^3^Centre for Nutrition and Food Sciences, The University of Queensland, St Lucia, QLD, Australia; ^4^Nutrition and Health, CSIRO, Adelaide, SA, Australia; ^5^Department of Molecular and Cellular Biology, College of Biological Science, University of Guelph, Guelph, ON, Canada; ^6^School of Agriculture and Food Sciences, Faculty of Science, University of Queensland, St Lucia, QLD, Australia; ^7^School of Science and the Tropical Medicine and Biology Platform, Monash University, Bandar Sunway, Malaysia

**Keywords:** starch synthase, amylopectin, amylose, rice, RNA silencing

## Abstract

The enzyme starch synthase IIa (SSIIa) in cereals has catalytic and regulatory roles during the synthesis of amylopectin that influences the functional properties of the grain. Rice endosperm SSIIa is more active in *indica* accessions compared to *japonica* lines due to functional SNP variations in the coding region of the structural gene. In this study, downregulating the expression of *japonica*-type SSIIa in Nipponbare endosperm resulted in either shrunken or opaque grains with an elevated proportion of A-type starch granules. Shrunken seeds had severely reduced starch content and could not be maintained in succeeding generations. In comparison, the opaque grain morphology was the result of weaker down-regulation of *SSIIa* which led to an elevated proportion of short-chain amylopectin (DP 6-12) and a concomitant reduction in the proportion of medium-chain amylopectin (DP 13-36). The peak gelatinization temperature of starch and the estimated glycemic score of cooked grain as measured by the starch hydrolysis index were significantly reduced. These results highlight the important role of medium-chain amylopectin in influencing the functional properties of rice grains, including its digestibility. The structural, regulatory and nutritional implications of down-regulated *japonica*-type *SSIIa* in rice endosperm are discussed.

## Introduction

Starch synthases (SS) play a critical role during starch biosynthesis, elongating glucan chains by the addition of glucose from the substrate ADP-glucose (Denyer et al., 1995; Denyer et al., 1996). These elongated glucan chains act as substrates for branching enzymes and debranching enzymes (Tetlow and Emes, 2014; Pfister and Zeeman, 2016). Cereals possess some isoforms of SS with varying substrate affinities and catalytic activities and of these, the endosperm-specific enzyme SSIIa has a particularly important role in starch biosynthesis. The gene for starch synthase IIa (*SSIIa*) codes for a major starch synthase enzyme isoform involved in the elongation of short-chain amylopectin in the cereal endosperm, which is important in distinguishing starch properties (Hannah and James, 2008; Jeon et al., 2010; Tetlow and Emes, 2017). In rice, the *SSIIa* gene [also known as *acl(t)*, *alk*, *gel(t)* and *SS2-3* gene in other studies] was originally mapped to the *alk* locus located on chromosome 6 (Umemoto et al., 2002; Gao et al., 2003; Umemoto et al., 2004) and is highly expressed during grain development (Hirose and Terao, 2004; Ohdan et al., 2005). *SSIIa* alleles determine the peak gelatinization temperature (GT) of rice, an essential trait in predicting cooking and eating qualities (Umemoto and Aoki, 2005; Waters et al., 2006).

Several single nucleotide polymorphisms (SNPs) located along the SSIIa coding gene have been linked with varietal differences in GT due to variations in amylopectin chain length distribution (CLD) (Umemoto et al., 2002; Umemoto and Aoki, 2005; Bao et al., 2006; Waters et al., 2006; Bao et al., 2009; Cuevas et al., 2010). Using the SSIIa sequence from the *indica* line Kasalath as the canonical protein sequence, previous research has demonstrated that substitution of either Valine-737 with Methionine (due to SNP3, which is G in Kasalath and A in Nipponbare at 2209 bp from the start codon), or Leucine-781 with Phenylalanine (due to SNP4, which is GC in Kasalath and TT in Kinmaze at 2340–2341 bp from the start codon) consequently lead to a reduction in specific activity of less than 10% compared to that of the *indica* sequence (Nakamura et al., 2005; Umemoto and Aoki, 2005). Substitutions of Methionine and Valine are common in the *japonica* lines. Thus, higher proportions of shorter amylopectin chain (S-type) is common among *japonica* rice lines such as Nipponbare because its SSIIa is weakly active, making the enzyme less efficient in catalyzing the elongation of short amylopectin chains, which leads to low GT (Umemoto et al., 1999; Nakamura et al., 2002, 2005; Umemoto and Aoki, 2005; Waters et al., 2006; Cuevas et al., 2010). In contrast, a higher proportion of longer amylopectin chain (L-type) is common among *indica* rices such as IR64 because its SSIIa enzyme is catalytically active (Nakamura et al., 2005). This results in elongation of short amylopectin chains and hence the increase in GT observed in the grain starch of *indica* rice accessions. Complementation of *japonica* S-type amylopectin (weakly active) in Nipponbare by *indica SSIIa* (active) from Kasalath produced *indica* L-type amylopectin (Nakamura et al., 2005).

Another potential consequence of amino acid substitutions due to SNP3 and SNP4 is on the ability of rice SSIIa to associate with starch granules (Umemoto and Aoki, 2005). Rice grains belonging to *japonica* types (haplotypes 3 and 4) have similar levels of SSIIa in the soluble phase but reduced levels in the starch associated protein fraction compared to those belonging to *indica* types (haplotypes 1 and 2) (Umemoto et al., 2004; Umemoto and Aoki, 2005; Waters et al., 2006; Bao et al., 2009). Furthermore, rice grains belonging to *indica* types are also observed to have higher amounts of starch-associated starch branching enzyme IIb (SBEIIb) compared to *japonica* types (Umemoto and Aoki, 2005) due to the SSIIa isoform present. This observation was confirmed by the association of *SSIIa* alleles with the relative distribution of SBEIIb and SSI between the starch granule and amyloplast stroma of rice (Luo J. et al., 2015). Additionally, following the multi-enzyme starch biosynthetic complex model in cereal endosperm proposed by Liu F.S. et al. (2012) and Tetlow et al. (2015), it is believed that SSIIa plays a scaffolding role in the formation of the complex (Umemoto et al., 2004; Nakamura et al., 2005; Umemoto and Aoki, 2005). Additionally, the presence of SSIIa appears to be important for the association of other proteins such as starch synthase I (SSI) and SBEIIb in starch granules (Liu F.S. et al., 2012). Clearly, therefore, SSIIa has diverse roles in starch biosynthesis by virtue of its enzymatic, scaffolding and stromal distribution functions during starch biosynthesis in cereals (Miura et al., 2018). All these findings highlight the importance of SSIIa in determining fine amylopectin structure and the resulting functional properties of rice grain.

In rice, an SSIIa mutant from the *japonica* line Kinmaze was identified completely devoid of *SSIIa* expression. This SSIIa null mutant, generated using *N*-methyl-*N*-nitrosourea, had a 4% increased amylose content, and a reduction of about 6°C in the gelatinization temperature (Miura et al., 2018). The 4% increase of amylose in the SSIIa null *japonica* rice resulted in the same levels of amylose comparable to that of indica rice lines. Loss of SSIIa in barley produced shrunken grains with high amylose and elevated resistant starch contents and reduced starch digestibility (Morell et al., 2003; Topping et al., 2003). It will be interesting to determine the phenotypic outcome of gradual reductions in the amount of weakly active SSIIa in rice grains with *japonica* background such as Nipponbare using transgenic approaches. Gradually reducing SSIIa expression by RNA silencing may aid in the further clarification of its roles during starch biosynthesis. In this study, the already low expression of weakly active *japonica*-type SSIIa in Nipponbare endosperm was further down-regulated to determine any impact on its possible roles in amylopectin biosynthesis and starch structure. Our results, using a different *japonica* rice line (Nipponbare) and a different technique (RNA silencing), are in broad agreement with the results obtained by Miura et al. (2018).

## Materials and Methods

### Downregulating *SSIIa* Expression

A hairpin RNA (hp-SSIIa) was constructed to downregulate the expression of SSIIa in the rice endosperm of *japonica* rice (Nipponbare cultivar) using a technique effective in partially downregulating the expression of *SBEIIb* in rice endosperm (Butardo et al., 2011). Total RNA was extracted from Nipponbare rice endosperm and used as the template for the synthesis of total cDNA (see next section). A 443 bp target sequence was amplified from the cDNA using forward (5′-GCTACCTCTGGGAGCTGAAGACGACGGAG-3′) and reverse primers (5′-GGGTGGGGTTCTCGGTGAAGA-3′) targeting position 1628-2071 of *japonica* rice SSIIa based on Nipponbare reference genome. The PCR fragment was cloned in pGEM-T Easy and subcloned into pBX17 and pVec8 using previously published methods (Butardo et al., 2011). The generated constructs were driven by wheat high molecular weight glutenin (wHMWG) promoter to ensure seed-specificity as previously demonstrated (Butardo et al., 2011). The generated constructs were verified by restriction digestion and DNA sequencing after every subcloning and transformation steps to ensure that the correct hairpin RNA sequences were maintained in the correct orientation. In addition, two artificial microRNAs (TTACAAAACAGAATCGTGGGC and TTAAGCGATATTATGTATCAC), also driven by wHMWG promoter were constructed using a technique which was previously shown to be more effective than hp-RNA in completely down-regulating the expression of SBEIIb in rice endosperm (Butardo et al., 2011). The Nipponbare rice calli were transformed with the silencing constructs using *Agrobacterium tumefaciens* AGL1 as previously described (Butardo et al., 2011). Tissue culture transformation, regeneration and selection were also as previously described (Butardo et al., 2011).

Regenerated transformed plants and negative controls were grown in pots partially submerged in water-filled tanks to simulate irrigated conditions inside a biosafety glasshouse. The temperature was maintained at 22°C during the night for 8 h and 29°C during the day time for 16 h of natural light. The actual average daily temperature was observed to be 26.5 ± 3.5°C and is consistent with our previous study (Butardo et al., 2011). Initial screening for putative transformants was done by PCR detection of a fragment of hygromycin resistance gene from genomic DNA of 1-month-old leaves extracted by FastDNA Kit (Q-BIOgene). The putative transformants were verified using gene-specific primers that amplify a hybrid PCR fragment containing a portion of the wheat high molecular weight glutenin (wHMWG) and a portion of the forward hairpin fragment. PCR amplification was carried out using HotStar Taq (Qiagen) using Hyper Ladder IV (Bio Line) as molecular weight standards.

### Gene Expression Analyses

RNA was extracted from grains at mid-development (15 dpa) using NucleoSpin RNA Plant (Macherey-Nagel). A total of 5 μg RNA template was used to synthesize cDNA using SuperScript III reverse transcriptase (Invitrogen). Quantitative real-time PCR (qRT-PCR) was done in a Rotor-Gene 6000 (Corbett) using 100 ng cDNA templates amplified utilizing previously published SSI and SSIIa primer pairs (Hirose and Terao, 2004; Ohdan et al., 2005; Yamakawa et al., 2007). The real-time PCR amplification was conducted using Platinum Taq DNA polymerase (Invitrogen) and Sybr Green I (Invitrogen) reporter dye. Comparative quantitation was conducted using α-tubulin as a reference gene (Toyota et al., 2006), with data validation and melt curve analysis done using the Real-Time Rotary Analyzer Software (Corbett).

### Protein Expression Analyses

Native soluble proteins from developing rice grains of selected homozygous plants (15 dpa) were extracted as previously described (Regina et al., 2006). This developmental stage ensures the maximal expression of *SSIIa* in wild-type rice grains, which was used to screen for SSIIa-downregulated lines. A total of 100 μg protein, quantified using Coomassie Protein Assay Reagent (Bio-Rad), was resolved in 4–10% precast gradient gels (Invitrogen). Total proteins were extracted from 5 to 20 dpa developing grains using a previously published method to track *SSIIa* expression across several developmental time points. In addition, soluble, granule-associated and granule-bound proteins from starch granules of mature grains (32 dpa) were also extracted as previously described (Butardo et al., 2012). For granule-bound proteins, 4 mg of starch for each sample was used for the extraction of proteins as described by Luo J.X. et al. (2015). Mature rice grains of selected SSIIa-downregulated lines were used to verify the silencing of the gene. Gels were blotted onto nitrocellulose membrane to detect SSI, SSIIa, and GBSSI using the appropriate antisera at 1:2000 dilution. Preliminary screening of SSIIa in total soluble protein extracts of T1 rice endosperms was conducted using anti-wheat polyclonal antibodies at 1:500 dilution but succeeding western blots for SSIIa were done using rice anti-SSIIa polyclonal antibodies due to improved specificity (Butardo et al., 2012). The immunoreactive proteins were probed by goat anti-rabbit or anti-mouse immunoglobulins conjugated to horseradish peroxidase (Bio-Rad). Antibody detection was carried out using ECL Western Blotting Detection Reagents (GE Healthcare Life Sciences) and Hyperfilm ECL chemiluminescence film (Amersham Biosciences). The film was developed using a CP 1000 automatic film processor (Agfa). Bands that were immunoreactive to anti-rice SSI and anti-wheat GBSSI antiserum were used to verify protein normalization in all Western blot experiments. Electroblotted gels were also stained with Sypro to verify the exact location of protein bands (see Supplementary Figure 2 as an example). IR64, an *indica* rice variety with catalytically active and highly expressed SSIIa were used as the positive control. This control rice line was grown with the Nipponbare transgenic and wild-type rice lines at the same time and in the same phytotron glasshouse to ensure uniformity of response.

### Grain Screening and Characterization

Only grains that have the hairpin RNA insert detected by PCR and downregulated SSIIa as detected by western blot as described above, were selected and subsequently characterized. Mature panicles of transgenic plants were harvested and dried at 37°C overnight. The opaque seeds were carefully threshed by hands, and then dehulled and polished by machine using standard methods. Shrunken seeds were also manually threshed and dehulled but they were not polished because they shatter into powder after passing through the milling machine. Consequently, all succeeding starch structural and functional analyses were done in polished (white) and wholemeal flours (brown) for opaque lines and only in wholemeal flours for shrunken lines. Photomicrographs of whole rice grain samples were obtained using either a LEITZ M8 stereomicroscope or by scanning in Image Scanner III (GE Healthcare Health Sciences). Some seeds were set aside for planting in subsequent generations.

### Carbohydrate and Digestibility Analyses

Peak GT from rice flour was measured by differential scanning calorimetry (Cuevas et al., 2010). Total starch content was determined using a 96-well plate format of a Megazyme assay procedure (AACC Method 76.13). The apparent amylose content (AAC) of flour samples was determined by iodine colorimetry using an AutoAnalyser3 Digital Colorimeter (Brann + Luebbe, United States). Data analysis was performed using the colorimeter’s Automated Analyser Control and Evaluation Software. Rice flour samples of IR24, IR64 and IR8 were used as amylose calibration standards and check controls.

The resistant starch (RS) content and estimated glycemic score (EGS) of freshly cooked polished rice grains were estimated using an *in vitro* starch hydrolysis index (HI) method which mimics the oral and gastrointestinal phases of carbohydrate assimilation in humans (Butardo et al., 2011). The HI method was comprehensively validated against *in vivo* clinical measurements in adult volunteers (Fitzgerald et al., 2011). A total of 50 and 500 mg of available carbohydrates were used to predict EGS and RS, respectively. For EGS prediction, aliquots of supernatant from starch hydrolyzates were sampled at designated regular time intervals for up to 5 h and glucose concentration determined using an automated electrochemical procedure (Butardo et al., 2011; Fitzgerald et al., 2011). Because the rice grains generated in this study were transgenic, which required special biosafety clearance for human consumption, the digestibility values obtained were used as highly correlated proxy measures for glycemic impact upon rice grain consumption based on previous work (Butardo et al., 2011; Fitzgerald et al., 2011).

### Starch Structural Determination

Determination of chain length distribution (CLD) of debranched amylopectin by fluorescence-assisted capillary electrophoresis (FACE) was performed based on a previous method (O’Shea and Morell, 1996). Molecular weight distribution (MWD) of debranched starch was determined by size-exclusion chromatography (SEC) as previously described (Castro et al., 2005). Precisely 10.0 mg of flour sample was gelatinized and debranched with isoamylase (Megazyme, Ireland) at 50°C. Immediately after debranching, samples were spun down using a microcentrifuge at room temperature. Aliquots for CE (50 μL) were obtained from the debranched supernatant and dried using a speed vacuum at 50°C for 2 h or until the pellet was completely dried. In addition, aliquots for SEC (750 μL) were obtained and desalted using AG 501-X8 (D) resin (Bio-Rad) for 30 min in 50°C water bath with occasional mixing by inversion every 10 min. Each dried pellet for CE analysis was labeled for 16 h with the 3.5 μL 0.2M 9-aminopyrene-1,4,6-trisulfonate (APTS) and analyzed in Beckman P/ACE system as previously described (Cuevas et al., 2010). On the other hand, 40 μL of each desalted sample was loaded into Alliance 2695 SEC machine (Waters, United States), resolved using an Ultrahydrogel 250 column (Waters, United States) using 0.05M NH4OAc pH 4.75 with 0.02% sodium azide as mobile phase and detected using 2414 Refractive Index detector (Waters, United States). For CE, the chain length distribution was determined from the peak area by converting to the velocity area to give *N(X)* (Demorest and Dubrow, 1991). For SEC, molecular weight distribution (MWD) was estimated from elution time using pullulan standards (Shodex P-82) calibrated with the Mark-Houwink-Sakaruda equation and universal calibration (Castro et al., 2005). A waxy rice was used to differentiate the debranched amylose and amylopectin regions with a cut-off at DP 120 (Butardo et al., 2011; Butardo et al., 2017). The following four MWD regions from debranched starch were identified: true amylose chains (DP > 1,000), long-chain amylopectin (DP 121-1000), medium-chain amylopectin (DP 37-120) and short-chain amylopectin (DP 6-36) as previously defined (Butardo et al., 2011; Butardo et al., 2017).

### Starch Granule Analyses

Cross-sections of transgenic rice grains that differed significantly from the controls were observed uncoated with an environmental scanning electron microscope (ESEM, Zeiss EVO LS15) under variable-pressure mode. Images of starch granules were taken with a back-scattered electron detector. Starch granules were also isolated and viewed under a polarized light microscope after iodine staining to check for birefringence. Granule size distribution (by volume) of the starch slurries was determined using a laser diffraction particle size analyzer (Mastersizer 2000, Malvern Instruments, Malvern, United Kingdom). The percentage of small starch granules was determined using a cut-off diameter of 1.9 μm (refer to the results section). The diameter of large starch granules (>1.9 μm) was calculated as the diameter of starch granules at the peak of large starch granules. Characterization of starch crystallinity by x-ray diffraction (XRD) was carried out on a Panalytical X’Pert Pro diffractometer using the crystal defect method based on our previous publication (Lopez-Rubio et al., 2008). Solid-state ^13^C cross polarization/magic angle spinning (CP/MAS) nuclear magnetic resonance (NMR) experiments were performed at a ^13^C frequency of 75.46 MHz on a Bruker MSL-300 spectrometer also as previously described (Butardo et al., 2011).

### Sampling and Statistical Analyses

Three biological replicates from at least two independent transformed lines were used for every analysis whenever applicable during the screening of hp-SSIIa-shr and hp-SSIIa-op. Mature grains of hp-SSIIa-op were used for whole grain and starch analyses for five generations (T1 to T5), planted and harvested at different seasons to assess stability and replicability of traits. Gene and protein expression profiling was performed on later generations (T3 to T5). A comparison of results was done using Nipponbare negative segregant as control. Statistical analyses (one-way analysis of variance with Tukey post-test, two-way analysis of variance with Bonferroni post-test, and unpaired *T*-test) were done using GraphPad Prism Version 6. The standard error of the mean (SEM) was used to representing error values. Statistical significance was defined at least as *P* < 0.05.

## Results

### Screening of Transgenic Lines

**FIGURE 1:**
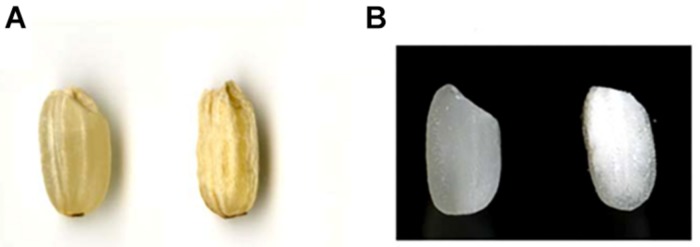
Seed morphology of representative grain samples harboring hp-SSIIa construct compared with the control. **(A)** Unpolished grain of a negative segregant (left) compared with hp-SSIIa-shr line (SS28), showing shrunken seed phenotype. **(B)** Polished grain of a negative segregant (left) compared with an hp-SSIIa-op line (SS5), showing opaque phenotype.

A total of 35 independent transformants were obtained from hp-SSIIa lines, of which 31 (88%) harbored the hygromycin resistance gene based on marker screening by PCR. A total of 5 out of 31 lines (16%) of hp-SSIIa were selected at T1 generation based on corroborative results from the seed appearance (Figure 1) and PCR screening using hygromycin resistance gene marker. Three of the selected segregating hp-SSIIa lines (9.6%) had shrunken seeds (SS9, SS17, SS28), while the other two lines (6.4%) had chalky to opaque seeds (SS3 and SS5). These selected lines were designated as hp-SSIIa-shr and hp-SSIIa-op, respectively, with representative seed samples shown in Figure 1. The hp-SSIIa-op grains are amenable to gentle polishing as they tend to fissure and break using standard milling methods. The two hp-SSIIa-op lines were viable in succeeding generations and they were stably maintained beyond T5 generation without losing the observed phenotypic traits. In contrast, grains of hp-SSIIa-shr cannot be polished because the shrunken seeds are brittle and shatter into powder during milling. In addition, the shrunken seeds of the hp-SSIIa-shr lines were either sterile or they did not fully mature in the succeeding generations. Those that grew at T2 stage had low germination rates and they needed to be grown by tissue culture. All the hp-SSIIa-shr lines were sterile beyond the T2 generation, with most spikelets empty or filled with transparent watery grain. As both the hp-SSIIa-shr and hp-SSIIa-op phenotypes were generated from multiple independent transgenic events, it is highly unlikely that the phenotype is due to the chance perturbation of a gene critical for the grain development. We demonstrate below that the phenotype is due to the reduction of SSIIa.

A total of 23 independent transformants were obtained that harbored artificial microRNAs targeted toward SSIIa (ami-SSIIa). Three ami-SSIIa lines cloned using the osa-miR528 backbone using the pWBVec8 expression vector were selected at T1 generation based on grain appearance and PCR screening. However, the lines could not be maintained beyond T2 generations. Consequently, the results for hairpin RNA downregulation of *SSIIa* expression in Nipponbare rice endosperm are the only ones reported in this study.

### Characterization of Starch Granules

**FIGURE 2:**
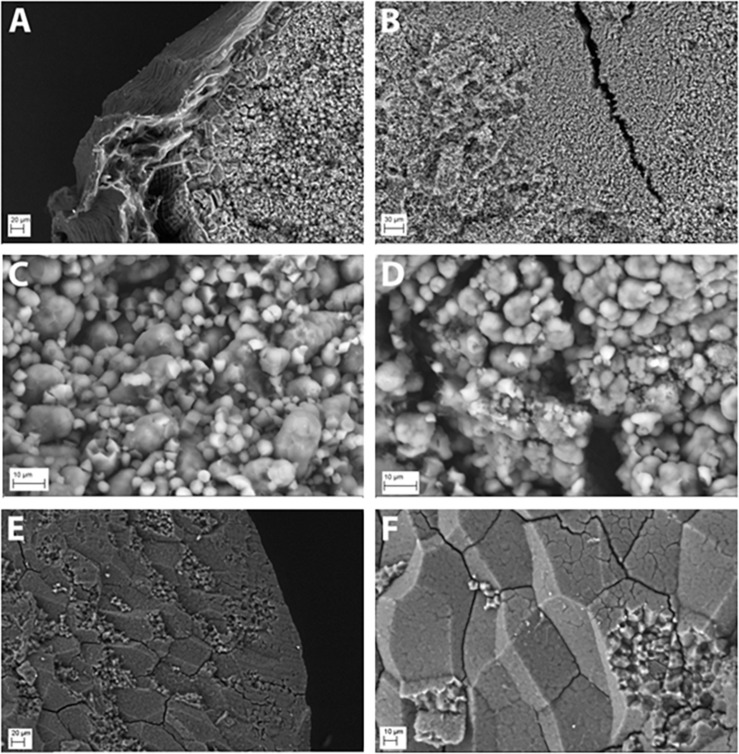
Starch granule morphology of representative hp-SSIIa-shr shrunken (SS28) and hp-SSIIa-op opaque (SS5) lines compared with the control. The starch granules of hp-SSIIa-shr, **(A)** showing the edge and **(B)** the middle section of an unpolished grain, demonstrating that severely distorted seed development is due to aborted starch granule formation. The starch granules of hp-SSIIa-op, **(C)** showing mixture of rounded compound and simple starch granules, **(D)** with some very small starch granules in the middle of the grain. The parent Nipponbare is used as a control, **(E)** showing the edge of a polished grain **(F)** and the middle showing compact and angular compound starch granules.

The starch granules of hp-SSIIa-shr T1 seeds were severely distorted during development compared with the controls. The granules were rounded and had lost their compound structure (Figure 2). Furthermore, the starch granules located toward the middle region of the grain were very small and their development appeared to be aborted (Figures 2A,B). Starch synthesis appears severely hampered based on seed morphology (Figure 1), starch granule appearance (Figures 2A,B) and reduced total starch content (Table 1). The starch granules of some less shriveled seeds have formed into a complex quaternary structure but they still lost their angularity (data not shown).

**TABLE 1:** Functional properties of hp-SSIIa lines compared with the controls.

Lines	Apparent Amylose (%)	Total Starch (%)	Peak GT (°C)	Resistant Starch (%)^∗∗^	Glycemic Index (predicted)
hp-SSIIa-shr^∗^	6.0 ± 0.8^a^	73.0 ± 2.1^a^	71.8 ± 0.0^a^	ND	ND
Nipponbare Brown	7.2 ± 0.0^a^	80.7 ± 1.4^c^	ND	ND	ND
hp-SSIIa-op	13.8 ± 0.6^b^	92.9 ± 2.4^b^	70.4 ± 1.1^a^	0.2 ± 0.1	63.5 ± 3.5^a^
Nipponbare Polished	15.2 ± 0.8^b^	90.4 ± 0.9^b^	73.9 ± 0.6^b^	0.2 ± 0.0	85.2 ± 1.2^b^

*^∗^Unpolished rice grains were used because shrunken grains are brittle and not amenable to polishing. ^∗∗^Using CSIRO Method. ND, not determined. Data marked with the different letters are significantly different at *P* < 0.05.*

**FIGURE 3:**
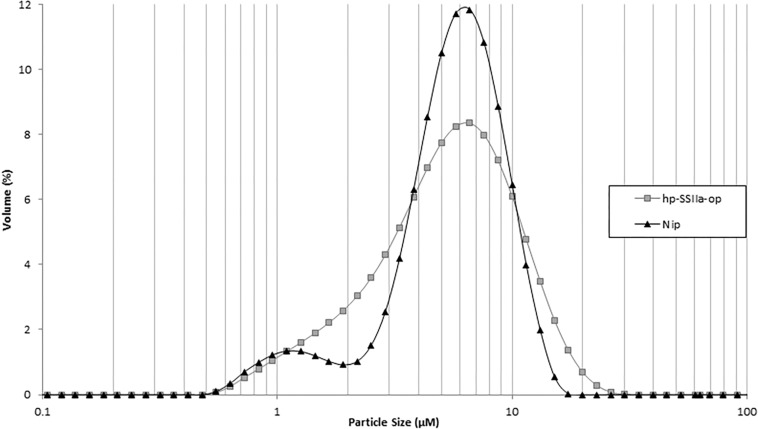
Particle size analysis of representative hp-SSIIa-op (SS5) in comparison with the control Nipponbare (Nip). The *y*-axis represents the amount of each size of starch granules as a percentage of total starch. The *x*-axis represents the size of starch granules. The names of lines are labeled on the right of the graph.

In contrast to the phenotypic consequence observed in hp-SSIIa-shr seeds, most of the starch granules of hp-SSIIa-op are rounded but they have maintained their compound structure (Figures 2C,D). The distribution of small starch granules in these opaque samples was reminiscent of the hp-SSIIa shrunken starch grain phenotype described above (Figures 2A,B). To provide more quantitative results, one line (SS5) was tested using a particle size analyzer which confirmed that it has a higher proportion of smaller starch granules compared to Nipponbare, approximately 1–4 μm in size (Figure 3). In addition, it also has higher proportion of bigger starch granules at 10–11 μm in size compared to its parental line. In contrast to the unimodal starch granule size distribution of hp-SSIIa-op, Nipponbare had a bimodal distribution, with a higher proportion of bigger starch granules 4–10 μm in size (Figure 3). T4 grains of hp-SSIIa-op (SS5) were obtained from the selfed panicles in order to verify that the observed starch granular organization was maintained. The small granules were still more pronounced toward the middle of the grain (data not shown).

The particle size distribution of starch granules in hp-SSII-shr was not determined due to the sample size limitation. However, based on the starch granule morphology (Figures 2A,B), it is expected that the proportion of its smaller-sized starch granules would be elevated even when compared to hp-SSIIa-op. Despite pronounced alterations in starch granule organization, the hp-SSIIa-op lines retained their A-type crystalline polymorph similar to its wild-type parent Nipponbare (Supplementary Figure 1). The crystallinity of hp-SSIIa-op as estimated by ^13^C CP/MAS NMR also did not vary significantly from that of Nipponbare (Supplementary Table 1). Lastly, all the starch granules exhibited normal birefringence similar to that of the control (data not shown).

### Characterization of Grain and Starch Functional Properties

Table 1 summarizes the results of the functional assays of hp-SSIIa lines compared with the controls. Analyses for hp-SSIIa-shr lines were done on unpolished T1 grains as they were brittle and shattered upon polishing, while the analyses of hp-SSIIa-op were done on polished grains for reasons described earlier, although they were more prone to over milling due to softer grains. The apparent amylose content of polished hp-SSIIa-op lines was not significantly different from that of the control. Similarly, the starch from hp-SSIIa-shr lines had percentage amylose comparable with that of unpolished control grains. The total starch content including the soluble glucan of hp-SSIIa-shr was reduced by 8% compared to Nipponbare brown rice control (*P*-value < 0.0001), while that of hp-SSIIa-op had no reduction compared to polished Nipponbare control. The peak gelatinization temperature of our rice lines are quite high compared to other reference values for Nipponbare in literature, which is 66.5°C in Umemoto et al. (2008), but closer to 68.0°C in Waters et al. (2006). We attribute the difference in GT values to variations in DSC machines and calibrations methods employed. Nonetheless, the peak gelatinization temperature of the shrunken and opaque lines of hp-SSIIa was reduced by at least 2°C compared to Nipponbare, and this was found to be statistically significant (*P*-value = 0.0320). Lastly, the resistant starch content of the hp-SSIIa-op lines was unaltered while the predicted glycemic index by *in vitro* hydrolysis index was significantly reduced (*P*-value = 0.0005). The hp-SSIIa-shr lines were not included in the estimation of resistant starch content and *in vitro* hydrolysis index due to sample size limitations and also because they were not amenable to polishing.

### Starch Structural Characterizations

**FIGURE 4:**
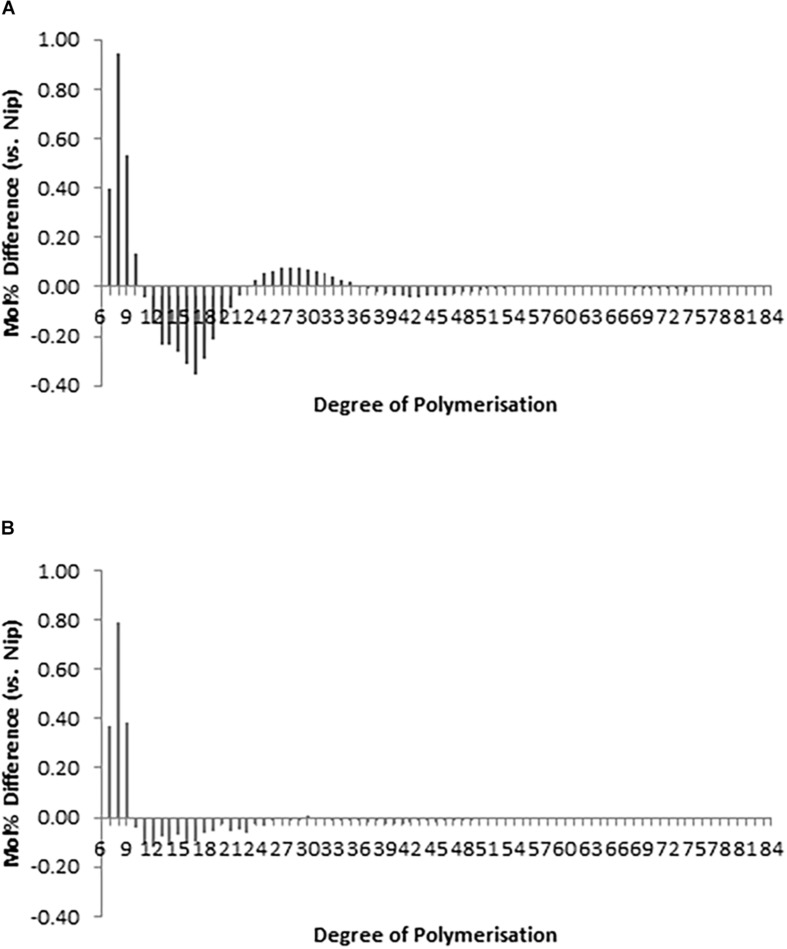
Chain length distribution (CLD) profile of debranched amylopectin from representative **(A)** hp-SSIIa-op (SS5) and **(B)** hp-SSIIa-shr (SS28) lines at T1 generation, showing significant elevation in short amylopectin A-chains from DP 6-10 and reduction from DP 12-24 (*P* = 0.0004). The results are average of three trials. Comparison of CLD is expressed as a difference plot (percentage of molar molecules) between each mutant and control Nipponbare.

**FIGURE 5:**
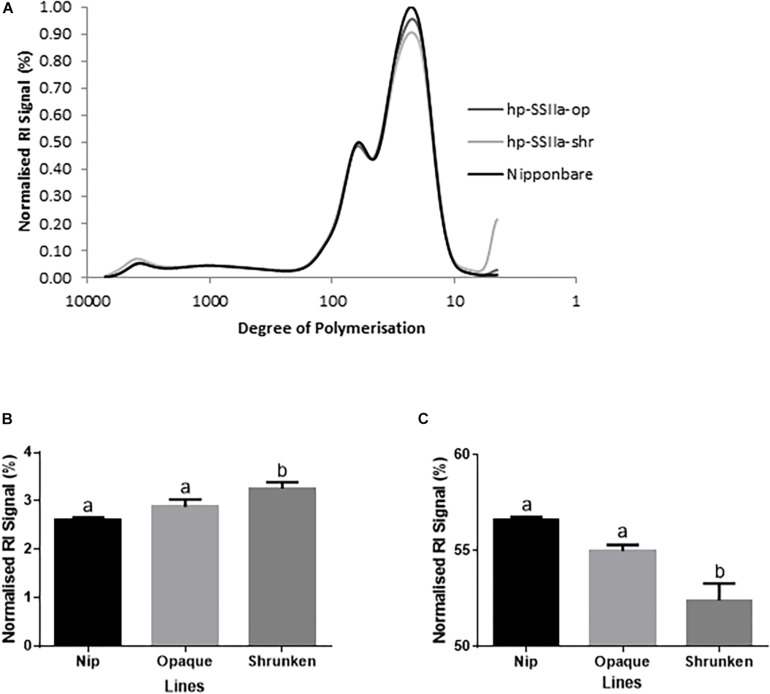
Molecular size distribution profile of debranched starch in T1 generation by SEC **(A)** for representative lines of hp-SSIIa-op (SS3 and SS5) and hp-SSIIa-shr (SS17 and SS28) compared with Nipponbare control. **(B)** Short chain amylopectin (DP 6–12) were elevated (*P*-value < 0.05) and **(C)** medium chain amylopectin (DP 13–36) were reduced (*P*-value < 0.02) in the transgenic lines compared to Nipponbare control. The profiles show the distribution of normalized refractive index (RI) signals of debranched starches from each sample. Mean comparison with control using Bonferroni multiple comparison test revealed that only the shrunken lines have statistically significant result. Bars with different letters indicate significantly different mean. The identity of samples is indicated on the right side of the figure.

The chain-length distribution (CLD) characterization of debranched amylopectin from selected seeds based on appearance, western blot and PCR screening of hp-SSIIa-op opaque lines (Figure 4A) and hp-SSIIa-shr shrunken lines (Figure 4B) revealed a significant increase in the proportion of short amylopectin chains (DP 6–12). In addition, a small decrease in longer chains of DP 12–24 was detected in both hp-SSIIa-op and hp-SSIIa-shr lines (Table 1). This CLD is corroborated by molecular size distribution analysis of normalized SEC traces from debranched starch which revealed significant elevation of short chains of apparent DP 6–12 in shrunken (3.2 ± 0.1 mol%) and opaque seeds (2.9 ± 0.2 mol%) compared with the control (2.6 ± 0.1 mol%) (Figures 5A,B). This shift in the proportion of short-chain amylopectin is accompanied by a significant reduction in medium-chain amylopectin of apparent DP 13–36 for shrunken (52.4 ± 1.7 mol%) and opaque seeds (54.9 ± 0.6%) compared with the control (56.6 ± 0.2%) (Figure 5C). There was no change observed in the proportion of true amylose chains (apparent DP > 1000) and long-chain amylopectin (DP 121-1000). Similar shifts in chain length and molecular size distribution profiles of amylopectin were further confirmed in homozygous generations of hp-SSIIa-op SS5 line at the T4 generation (data not shown).

### Determination of SSIIa Protein Inside Starch Granules

**FIGURE 6:**
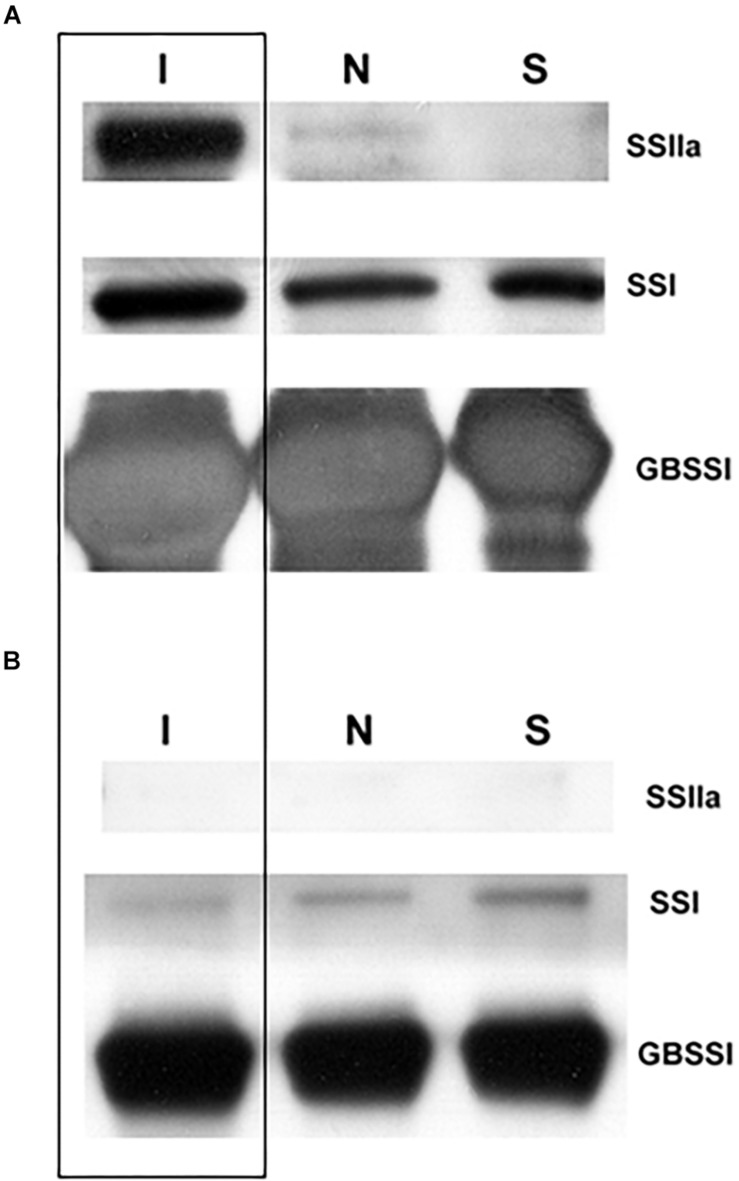
Western blot analysis of SSIIa, SSI and GBSSI in **(A)** granule-bound and **(B)** soluble proteins of hp-SSIIa-op (SS5) (S) compared with its parent Nipponbare (N) and an *indica* positive control IR64 (I) (boxed) using mature grains.

T4 grains of hp-SSIIa-op SS5 were further characterized to determine whether SSIIa was detectable inside the starch granules after *SSIIa* downregulation by hairpin RNA silencing. Western blot detection of granule-bound proteins of SS5 revealed that SSIIa was undetectable (Figure 6A). In contrast, a small amount of SSIIa was detected in Nipponbare, while its concentration in the *indica* control (IR64) was very high. As expected, the amount of GBSSI was higher in IR64 compared to Nipponbare and SS5, while SSI appeared to be similar in the granule-bound proteins of the three lines tested. SSIIa was not detected in the soluble protein fraction from flour samples of mature desiccated grains of the three lines tested (Figure 6B). The amount of SSI appeared similar in the three lines tested, indicating proper protein normalization (Supplementary Figure 2). The amount of SBEIIb and GBSSI inside the starch granules appeared to be higher in IR64 compared to that of the other two lines (Supplementary Figure 2).

**FIGURE 7:**
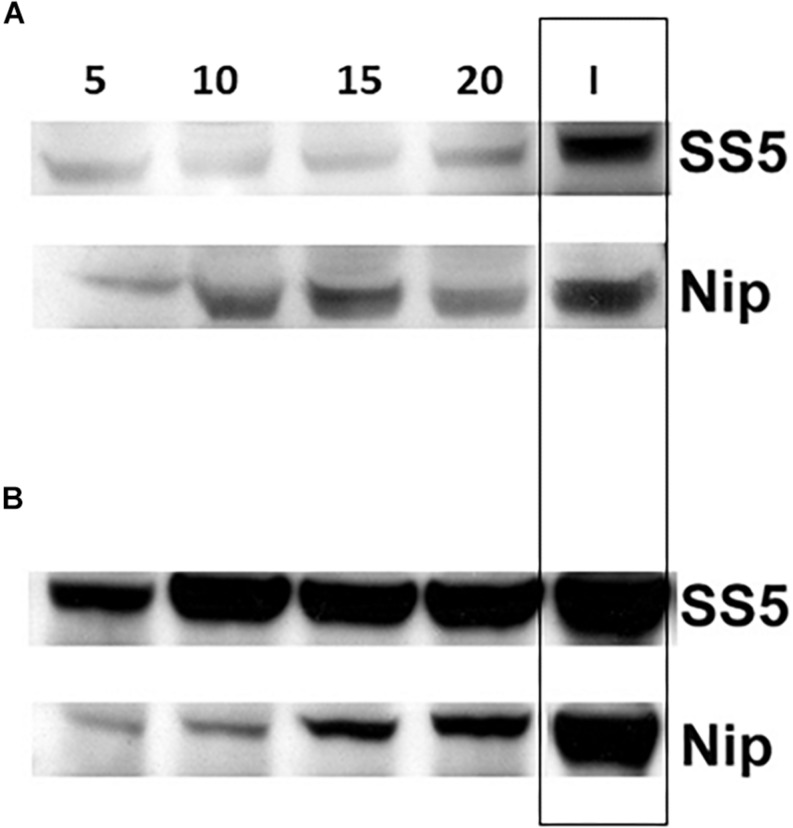
Western blot analysis of **(A)** SSIIa and **(B)** GBSSI protein expression in the total protein extract from developing grains of hp-SSIIa-op (SS5) compared with Nipponbare (Nip) using 5–20 DPA grains. 15 DPA IR64 (I) was used as a reference positive control band (boxed). Days of each sample is labeled above the lanes (5, 10, 15, and 20).

Comparison of the amounts of SSIIa present in the total protein extracts of developing endosperms of SS5 (5, 10, 15, 20 dpa) confirmed that the level of SSIIa in the total protein extract was partially down-regulated compared to their corresponding developmental stages of Nipponbare control, as well as compared to the 15 dpa IR64 used as a reference band (Figure 7A). The amount of GBSSI in SS5 appeared up-regulated compared to Nipponbare (Figure 7B). Western-blotting analyses (Figure 6B) and transcript analyses of 15 dpa grains by qRT-PCR (Supplementary Figure 3) revealed that the amount of SSIIa in SS5 was half of that in Nipponbare, and the amount of SSI was two-fold of that in Nipponbare.

## Discussion

Our results revealed that endosperm down-regulation of *japonica*-type SSIIa resulted in distinct seed and starch granule morphologies, elevation in the proportion of short-chain amylopectin, and the reduction of peak gelatinization temperature and estimated glycemic score of cooked grains. Nipponbare is a *japonica* variety with an SSIIa belonging to haplotype 3 (Waters et al., 2006). This *japonica* variety is believed to have a weakly active SSIIa enzyme (Umemoto et al., 2004; Nakamura et al., 2005). However, functional genomic validation presented here showed that the downregulation of SSIIa in Nipponbare led to pronounced changes in seed appearance, starch granule morphology, amylopectin fine structure, as well as thermal and digestibility properties (Table 1). These results demonstrate that SSIIa in Nipponbare either has a critical residual catalytic activity or possesses additional functions that are affected at these low concentrations as will be elaborated below.

### Effect of Downregulating SSIIa on Starch Granule Structure and Organization

The proportion of debranched amylopectin at DP 13-36 was further reduced in the SSIIa down-regulated lines compared to Nipponbare. It is highly likely that the alteration in seed appearance, starch granule morphology, and starch granule size distribution are the phenotypic consequences of this perturbation in amylopectin structure. These observations are in agreement with previous studies in null SSIIa mutants of wheat, barley and maize (Liu F. et al., 2012; Luo J. et al., 2015). The endosperm starch from these SSIIa-null mutants all exhibited increased proportions of short amylopectin chains (approximately DP 6–12) and decreased levels of medium chains (approximately DP 13–36) compared to their respective parents. Similar changes in the chain length distribution profile were observed in Nipponbare compared with NILs(*Alk*), a near-isogenic line of Nipponbare with introgressed *indica SSIIa* (Haplotype 1) from Kasalath (Umemoto et al., 2004) and a recent study of an SSIIa null mutation in *japonica* rice (Miura et al., 2018). The 8% reduction observed in the total starch content of the shrunken lines and the slight reduction in peak gelatinization temperature (at least 2°C) of all shrunken and opaque lines tested also support the hypothesis that the residual SSIIa in Nipponbare has functional roles because its downregulation leads to significantly altered starch biosynthesis in the rice grain. The reduction of the peak gelatinization temperature was consistent with the earlier report by Miura et al. (2018), which observed a reduction of about 6°C in the gelatinization temperature of the null SSIIa line.

SSIIa is involved in the formation of multi-enzyme complexes as demonstrated in other cereal endosperms where it may provide a central structural scaffold for other enzymes participating in amylopectin biosynthesis (Liu F.S. et al., 2012). More recent work has shown that similar protein complexes occur in rice (Crofts et al., 2015; Hayashi et al., 2018). Although SSIIa is enzymatically less active in *japonica* lines, and expressed at lower levels compared to *indica* lines, it may still be crucial for starch biosynthesis by providing the structural scaffold for the formation of enzyme complexes (Tetlow et al., 2015). Our results are consistent with the conclusion that the formation of the scaffold is affected when the expression of SSIIa is abolished (probably in the case of hp-SSIIa-shr) or when the amount of SSIIa is significantly reduced (as in the case of hp-SSIIa-op).

It is probable that the opaque seed phenotype is due to a milder SSIIa down-regulation, and that the more severe shrunken seed phenotype showed the further impact of the complete loss of SSIIa. We speculate that RNA silencing of SSIIa in rice endosperm is lethal to rice grain development due to pleotropic effects not detected in this study because it is accompanied by severe distortion of seed and starch granule morphology and reduction of starch content, which ultimately result in non-viable seed in succeeding generations. Unfortunately, it was not possible to directly test the expression of SSIIa in the homozygous shrunken lines due to sterility in succeeding generations. The three SSIIa down-regulated lines with shrunken grains (SS9, SS17, and SS28) were shown to have severely distorted starch granules whose formation appeared to have been aborted early during grain development. These lines also showed reduced total starch content and lower proportions of amylopectin chains of DP 12–36. These results suggest severely reduced activity of other enzymes involved in amylopectin biosynthesis upon reduction of SSIIa protein, and will be the focus of future studies. Such pleiotropic effects are consistent with the generally accepted idea of functional multi-enzyme complexes carrying out starch biosynthesis in cereals, which can be disrupted in the complete absence of SSIIa. Interestingly, the effects of down-regulation of SSIIa may be different in different genetic backgrounds, or under different environmental or growth conditions. A similar study showed field-grown plants lacking SSIIa able to flower and set seed (Miura et al., 2018). The study by Miura et al. (2018) also noted increases in apparent amylose, which were not observed in the present study. Further experiments need to be conducted to elucidate the factors responsible for causing the differences in growth and seed viability between the two SSIIa-deficient *japonica* rice lines.

The NMR data showed very similar levels of molecular order (double helix and single helix contents) between isolated granules from Nipponbare and the two hp-SSIIa lines tested. Double helical molecular order arises from non-enzymic inter-twining of adjacent glucan chains in the branched amylopectin structure, provided they are longer than about six residues (Gidley and Bulpin, 1987). It cannot, however, be determined which chain lengths are directly responsible for molecular order, and a change in branch length profile may or may not lead to a change in the intertwining of chains depending on the detailed architecture of the amylopectin molecules. It is clear, however, that even slight perturbations in amylopectin structure can influence starch granule packing, which in turn can have an impact on the translucency of the grain. This observation is supported by *SBEIIb* transgenic mutants in rice, which shifted from A to C to B-type crystalline polymorph and is accompanied by chalky to opaque grain phenotypes (Butardo et al., 2011).

### Effect of Down-Regulating SSIIa on Grain Starch Digestibility

Down-regulating the expression of *SSIIa* in *japonica* line, Nipponbare did not produce high amylose and high resistant starch phenotype as occurs in barley (Morell et al., 2003). The increase in amylose and reduced starch content in the SSIIa mutant in barley is accompanied by a significant increase in resistant starch and non-starch polysaccharide contents, as well as substantial reductions in total starch content and glycemic index (Morell et al., 2003; Topping et al., 2003). Another noteworthy SSIIa phenotype is found in the *sugary-2* mutation in maize where amylose levels doubled from ∼20% to ∼40% (Zhang et al., 2004). It is possible that down-regulating the expression of *SSIIa* in Nipponbare did not produce a high amylose phenotype because it is in a *Wx*^b^ background, where the GBSSI allele has low transcriptional efficiency (Sano, 1984; Hirano et al., 1998). More recently, a null mutation of SSIIa in Kinmaze (*japonica*) lead to a 4% elevation of amylose content in the *japonica* background (Miura et al., 2018). Considerable amounts of SSI and SBEIIb remain in the starch granule fraction of endosperms, although *SSIIa* has been significantly down-regulated, which was also reported by Miura et al. (2018). Our previous study on *SSIIa* mutants of different cereals suggested that only when SSI, SSIIa, and SBEIIb are all absent in the starch granules, the amylose content will be significantly elevated (Luo J. et al., 2015). It would be interesting to determine in future studies whether down-regulating or genome editing of *SSIIa* in *indica* lines where GBSSI is more active can lead to elevated amounts of amylose or long-chain amylopectin and whether simultaneously down-regulating *SSI*, *SSIIa* and *SBEIIb* lead to high amylose rice grains even in *japonica* background. However, it has been reported that null mutations of *ss1* and *be1*, or *ss1* and *be2b* resulted in sterile rice plants (Abe et al., 2014).

Interestingly, despite the lack of elevation in amylose and resistant starch contents, downregulating the *SSIIa* expression in Nipponbare reduced the predicted GI value, which is similar to the SSIIa null mutant in barley (Topping et al., 2003). The estimated glycemic score as measured by *in vitro* starch hydrolysis index (HI) was significantly reduced from 85 to 63 GI units (or 25%) in two hp-SSIIa-op lines (SS3 and SS5) based on biologically replicated assays. This reduction was still not as much as the reduction in glycemic index estimate for the ami-SBEIIb down-regulated lines (44 ± 1). Nonetheless, it is still noteworthy that the slight reduction in medium-chain amylopectin content in the hp-SSIIa-op line was linked with a lower glycemic index estimate even though this was not accompanied by an increase in amylose chains or long-chain amylopectin as was previously observed in the SBEIIb mutant line, IR36ae (Butardo et al., 2012) and down-regulated lines harboring ami-BEIIb (Butardo et al., 2011). Two candidate genes in rice for improving the GI response have been identified so far: SBEIIb (Butardo et al., 2011) and GBSSI (Fitzgerald et al., 2011). It appears that fine-tuning of amylopectin structure by SSIIa down-regulation provides a novel mechanism of reducing digestibility in rice by introducing subtle reductions in the proportion of medium-chain amylopectin (this study). Lowering the glycemic index without increasing the amylose and resistant starch contents is important in producing slowly digestible rice grains with acceptable cooking and eating qualities (Butardo et al., 2017). It is also possible, however, that other changes in the starch granules or non-starch constituents of the grain, which were not tested in this study, are responsible for the observed glycemic effects. For instance, the interaction of seed storage proteins and lipids with starch in rice can have a profound influence on grain digestibility (Butardo and Sreenivasulu, 2016). Other structural carbohydrates that act as dietary fiber such as hemicelluloses can also have an impact on digestibility (Butardo and Sreenivasulu, 2016).

In summary, down-regulating the expression of *SSIIa* in Nipponbare produced rice grains with distinct phenotypes, including alterations in seed appearance, starch granule morphology, starch granule size distribution, and amylopectin fine structure. Unlike barley, further reduction in the amount of SSIIa was not accompanied by an increase in the amylose and resistant starch contents in a *japonica* background. However, a slight reduction in the proportion of medium-chain amylopectin (DP 13–36) in the SSIIa down-regulated lines is associated with a reduction in the glycemic index estimates. Thus, in addition to increasing amylose content and increasing the proportion of long-chain amylopectin, a reduction in the proportion of medium chains of amylopectin is also associated with the reduction of GI. Lastly, this study also supports the data of Crofts et al. (2015) and Hayashi et al. (2018), which indicate the structural and regulatory role of SSIIa in multimeric enzyme complex formation in amylopectin biosynthesis in developing rice endosperms.

## Data Availability Statement

All datasets generated for this study are included in the article/Supplementary Material.

## Author Contributions

VB conducted all the genetic engineering, functional genomic validation, gene and protein expression, and starch biochemistry experiments. JL conducted the protein expression experiment of developing grains. ZL determined the size distribution of the starch granules. MG determined the crystallinity and x-ray diffraction patterns of starch granules. AB determined the estimated glycemic score of transgenic and control lines. SR assisted in rice tissue culture and transformation experiments. MG, IT, MF, SJ, and SR provided technical supervision, intellectual guidance, and multi-disciplinary expertise during the conduct of all experiments. VB wrote the manuscript with the assistance of all co-authors, especially IT and SR who helped in the several revisions of the manuscript.

## Conflict of Interest

The authors declare that the research was conducted in the absence of any commercial or financial relationships that could be construed as a potential conflict of interest.
