# Supplementary material for: Functional Genomic Validation of the Roles of Soluble Starch Synthase IIa in Japonica Rice Endosperm
Source: Front Genet. 2020 Apr 2;11:289. doi: 10.3389/fgene.2020.00289 (PMC7142255; doi:10.3389/fgene.2020.00289)
Supplement: Supplementary file 1 [file Data_Sheet_1.docx]

Supplementary Material

# Supplementary Table

**Supplementary Table 1**. Determination of crystallinity of hp-SSIIa-op by ^13^C CP/MAS NMR

| **Sample Code** | **Classification** | **% Double Helix**  **(±1)** | **% Single helix**  **(±0.5)** | **% Non-ordered**  **(±2)** | **Crystalline polymorph** |
| --- | --- | --- | --- | --- | --- |
| SS5 | hp-SSIIa-op | 37 | 2 | 61 | A |
| SS3 | hp-SSIIa-op | 33 | 2 | 65 | A |
| Nipponbare | wild type | 34 | 4 | 62 | A |

# Supplementary Figures

**Supplementary Figure 1**. X-ray diffraction pattern of hp-SSIIa-op SS5 (hp-SSIIa) displaying A-type crystalline polymorph similar to Nipponbare. Data are offset for clarity.


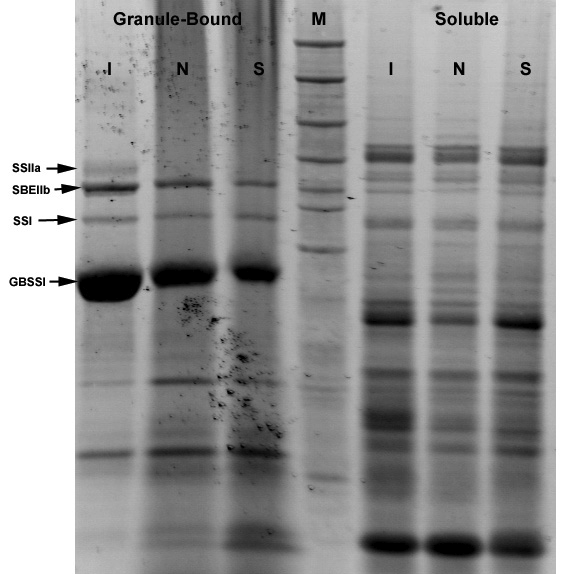


**Supplementary Figure 2**. Visualisation of granule-bound and soluble proteins in hp-SSIIa-op (SS5) (S) compared to its parent Nipponbare (N) and IR64 (I). Major protein bands are labelled based on BenchMark unstained protein ladder (M).

**Supplementary Figure 3**. Expression of (A) SSI and (B) SSIIa at 15 dpa of Nipponbare compared to hp-SSIIa opaque (SS5) transgenic line.
